# Optimization of the Effects of Different Temperatures and Compositions of Filmogenic Solution on *Lactobacillus salivarius* Using Predictive Mathematical Models

**DOI:** 10.3390/foods10010025

**Published:** 2020-12-23

**Authors:** Shênia Santos Monteiro, Wilton Pereira da Silva, Shirley Santos Monteiro, Josivanda Palmeira Gomes, Emmanuel Moreira Pereira, Alexandre José de Melo Queiroz, Rossana Maria Feitosa de Figueirêdo, Ana Paula Trindade Rocha, Hanndson Araujo Silva, Leyllanne Renalle Batista de Almeida, Mábia Ruana Silva de Sena, Antônio Gilson Barbosa de Lima

**Affiliations:** 1 Department of Agricultural Engineering, Federal University of Campina Grande, Campina Grande 58428-830, Brazil; shenia-monteiro@hotmail.com (S.S.M.); shirley_pinto_monteiro@hotmail.com (S.S.M.); josivanda@gmail.com (J.P.G.); alexandrejmq@gmail.com (A.J.d.M.Q.); rossanamff@gmail.com (R.M.F.d.F.); ana_trindade@yahoo.com.br (A.P.T.R.); hanndson@gmail.com (H.A.S.); leyllaalmeida@gmail.com (L.R.B.d.A.); mabia_ruana@hotmail.com (M.R.S.d.S.); antonio.gilson@ufcg.edu.br (A.G.B.d.L.); 2 Department of Agriculture, Federal University of Paraiba, Bananeiras 58220-000, Brazil; emmanuel16mop@hotmail.com

**Keywords:** fermentation, inulin, predictive modelling, probiotic

## Abstract

It is well known that intake of probiotic brings health benefits. Lactic bacteria with probiotic potential have aroused the interest of the industry in developing food products that incorporate such benefits. However, incorporating probiotic bacteria into food is a challenge for the industry, given the sensitivity of probiotic cultures to process conditions. Therefore, the objective of this study is to evaluate gelatin- and inulin-based filmogenic solutions as a potential vehicle for incorporating probiotics into food products and to model the fermentation kinetics. *L. salivarius* (*Lactobacillus salivarius*) growth in filmogenic solutions was analyzed under the influence of a variety gelatin concentrations (1.0–3.0%) and inulin concentrations (4.0–6.0%) and fermented under the effect of different temperatures (25–45 °C). A full 2^3^ factorial plan with three replicates at the central point was used to optimize the process. The impacts of process conditions on cell development are fundamental to optimize the process and make it applicable by the industry. The present study showed that the optimal conditions for the development of probiotic cells in filmogenic solutions are a combination of 1.0% gelatin with 4.0% inulin and fermentation temperature of 45 °C. It was observed that the maximum cell growth occurred in an estimated time of about 4 h of fermentation. *L. salivarius* cell production and substrate consumption during the fermentation of the filmogenic solution were well simulated by a model proposed in this article, with coefficients of determination of 0.981 (cell growth) and 0.991 (substrate consumption).

## 1. Introduction

*Lactobacillus* spp. is one of the most widely used probiotics in the lactic acid bacteria group and can be found in a wide variety of food products worldwide. This genus plays a very important role in food fermentation and can also be found in the gastrointestinal system of humans and other animals, in varying amounts, depending on the species, age of the host or location within the intestine [1]. In recent years, *Lactobacillus salivarius* has gained attention from researchers as a promising probiotic species. Probiotic properties, such as the ability to modulate the microbiota, produce antimicrobial substances, stimulate the protective immune response, inhibit fecal enzymatic activity and produce short-chain fatty acids, allowing a convenient acidification of the intestine, have been attributed to *L. salivarius* [2].

Due to the sensitivity of probiotics to common processing conditions such as heat treatment, low pH environments, high osmotic pressure and high redox potential, the design of effective physicochemical barriers to stabilize organisms is essential for their complete commercial exploitation in a wide variety of foods [3].

The products commonly found on the market as vehicles for probiotics are fermented milks and dairy products. However, fruits and vegetables have been studied as an alternative to the consumption of milk products. To ensure the maximum viability of probiotic cells in products of plant origin, drying techniques such as spray drying and freeze drying are the most applied for the manufacture of new products. As an alternative to increase the variety of probiotic products, the application of edible film added with probiotic cultures has been studied, and a large number of applications have already been investigated [3]. These investigations include bakery products [4], meat and fishing products [5], in addition to fruits [6] and cereal bars [7]. Although the results obtained are promising for application in food, the effect of process conditions, such as temperature, which influences process time, requires further research.

The prospects for using edible films as potential carriers of active ingredients are high. Edible films have the potential to stabilize food structures in multiple scale lengths, while creating bespoke structures (enhanced mechanical properties, extended shelf life, maintaining structural integrity), and can be used to provide nutritional improvements through the inclusion of probiotics [3].

Edible films used in food have different properties depending on the structural material. The optimization of the composition of edible films is one of the most important stages of research in this field, as they must be formulated according to the properties of the fruits and vegetables to which they must be applied [8], as well as the material of interest that one seeks to preserve, such as probiotic cells.

Predictive models of microbial interaction can help clarify how specific conditions that prevail in the food environment influence the effectiveness of the growth of lactic acid bacteria and/or their production of metabolites [9]. According to Whiting [10], predictive models describe the growth of microorganisms quickly, efficiently and economically, compared to traditional enumeration methods, which are costly and time-consuming. Mathematical models used in predictive microbiology are categorized mainly as primary, secondary and tertiary models. Primary models are mathematical equations that define growth data as a function of time under a constant environmental condition. The Baranyi model [11] is the most widely used primary model to describe microbial growth data. Secondary models use parameters determined by the primary models to predict changes in microbial structure and specific maximum growth rate as a function of environmental factors such as temperature, oxygen, pH and water activity. The maximum specific growth rate, which is one of the most critical kinetic growth parameters, can be modeled using the secondary models. Temperature plays the fundamental role of affecting the growth behavior of microorganisms in food. The Ratkowsky model [12] and Arrhenius model [13] are examples of secondary models often used to determine temperature dependence in microbial growth. Tertiary models are formed by the combination of primary and secondary models and use a computer as an estimation tool, but these models suffer from the lack of experimental information in relation to many specific foods [14].

Several mathematical models have been used in the literature to describe experimental growth data over time. As an example, Germec et al. [15] modeled lactic acid fermentation in a bioreactor with carob extract. To determine the modeling success, root mean square error (RMSE), mean absolute error (MAE), determination coefficient (R^2^), bias factor (BF) and accuracy factor (AF) were used. Ilgin et al. [16] studied inulinase production and mathematical modeling from carob extract by using *Aspergillus niger*. In order to define the best models (Baranyi and Cone model), the authors used the following statistical indicators: RMSE, MAE, BF and AF. Silva et al. [17], when modeling the growth of *Lactobacillus viridescens* under non-isothermal conditions in sliced and vacuum-packed ham, compared the values predicted by the Baranyi model with the experimental values using the following statistical indicators: RMSE, BF and AF. Tarlak et al. [14], when fitting the Baranyi model to determine the kinetic growth parameters of *Pseudomonas* spp. on button mushrooms stored under isothermal and non-isothermal conditions, used RMSE and R^2^ as indicators of model fit quality. In general, studies involving cell growth and substrate consumption available in the literature do not evaluate each parameter of mathematical models through Student’s *t*-test, which makes it possible to determine whether a parameter can be considered equal to zero, despite having the value obtained in the fit. For this, it is necessary to know not only the value of the parameter, but also its uncertainty and the number of degrees of freedom of the fitting with which the parameter was obtained. Although the use of this test is fairly common in studies involving experimental planning, perhaps due to the large number of parameters involved in an analysis, this test is not as common in the fitting of more compact functions to an experimental dataset. However, this test makes it possible to evaluate, in any of the two situations mentioned above, whether the calculated parameters have (or not) statistical significance. In addition, it is appropriate to highlight the importance of using predictive models to describe the growth of probiotic microorganisms in edible films with potential for application in foods, especially because they are considered as innovative products. In this context, the objectives of this article are defined below.

The aim of this study is to describe and evaluate the interaction of the composition of filmogenic solutions on the growth of *L. salivarius* under different temperatures and to describe the fermentation process using predictive microbial interaction models. The model for cell growth and substrate consumption were chosen, the usual statistical indicators in this field of study (R^2^, chi-square, χ^2^, BF and AF), in addition to considering the Student’s *t*-test.

## 2. Materials and Methods

### 2.1. Preparation and Inoculation of L. salivarius in the Filmogenic Solution

The choice of gelatin and inulin for the preparation of filmogenic solutions is due to the objective of combining the functional properties of each material, seeking to obtain an efficient filmogenic solution as a gas barrier and good mechanical properties at low relative humidity [18]. Gelatin has technological properties well known in the industry, one of which is encapsulation, capable of protecting the material of interest from adverse conditions, such as exposure of the material to high temperatures. Combined with gelatin, inulin aggregates the film-forming solution with gelling properties depending on the concentration, among other factors, in addition, inulin is a functional additive relevant to the food industry due to its prebiotic properties, promoting the growth of probiotic bacteria such as *L. salivarius*.

The concentrations of gelatin (1.0, 2.0 and 3.0%) and inulin (4.0, 5.0 and 6.0%) in the preparation of filmogenic solutions, as well as the effects of fermentation temperature (25.0, 35.0 and 45.0 °C) were studied through a full 2^3^ factorial plan with 3 replicates at the central point. The specifications of the conditions for each of the 11 experiments were defined in Table 1.

Effects of independent variables on the physicochemical and biological characteristics of *L. salivarius* were analyzed using the response surface methodology, which assumes that there is a polynomial function that relates each response of interest to the independent variables. The statistical test of analysis of variance (ANOVA) was performed with a 95% confidence level, including the statistical significance of each term of the model to be fitted (*p*-value), the estimated effects on each term and the coefficient of determination of the model in order to establish its reliability, using Statistica 7.0 software (7.0.61.0, StatSoft Inc., Tulsa, OK, USA) [19].

The mixtures of gelatin and inulin were heated under shaking at 80 °C until complete solubilization of gelatin and inulin in water. The heating of the solution, in addition to increasing the solubility of gelatin and inulin, aims to inactivate possible pathogenic microorganisms. The filmogenic solution was cooled to temperatures indicated in the full factorial plan for the inoculation of in freeze-dried probiotic culture. The lyophilized probiotic strain of *L. salivarius* was inoculated in the filmogenic solutions at an initial concentration of 10 LogCFU·mL^−1^ and shaken for 10 min to obtain homogeneity in the sample. The initial concentration of 10 LogCFU·mL^−1^ was adopted in order to obtain a maximum concentration of probiotic cells in the filmogenic solution capable of promoting benefits to the consumer’s health, when consumed through application in food. It was taken into account that, regardless of the process used in the production of food coated with the probiotic filmogenic solution, there may be a reduction in cell viability.

To study the kinetics of the fermentation process, a 165 mL volume of probiotic filmogenic solution was used in fermentation, and this volume was distributed in 11 15 mL falcon tubes. The fermentation process occurred under isothermal conditions, and for this, a BOD (Biochemical Oxygen Demand) incubator with controlled temperature was used, without shaking. The biological characteristics of *L. salivarius* in filmogenic solutions were determined by monitoring fermentation for 20 h. At each interval of 2 h, a 15 mL aliquot of the probiotic filmogenic solution was collected, and then, the cell concentration was determined by the direct counting method in Neubauer chamber. The pH was monitored by taking each measurement with a pH meter; total soluble solids were determined using a portable refractometer, and reducing sugars were determined by the calorimetric method using the 3,5-dinitrosalicylic acid, according to Miller [20]. The contents of reducing sugars in probiotic filmogenic solutions during fermentation were used to describe substrate consumption during the fermentation process with *L. salivarius*. Although the experiments related to cell growth kinetics and substrate consumption were carried out for 20 h, only the data collected in the first 14 h were analyzed, eliminating the period referring to the decline or cell death phase.

### 2.2. Mathematical Models: Cell Growth and Substrate Consumption

The average data of cell growth and substrate consumption obtained during the fermentation stage of the experiments were used for mathematical modeling of kinetics. The study of mathematical models was carried out to describe the process of *L. salivarius* growth and substrate consumption in the probiotic filmogenic solution that had the greatest productive advantage. In addition to Statistica software, LAB Fit Curve Fitting Software (www.labfit.net) was also used in this study of kinetics. Initially, primary models were selected from the literature to describe cell growth and substrate consumption in the probiotic filmogenic solution, as described below.

#### 2.2.1. Baranyi Model

The Baranyi model [11], with 5 parameters, was one of the models used to estimate the kinetic parameters of *L. salivarius* growth in filmogenic solutions under isothermal conditions and to predict substrate consumption. This model is given according to Equation (1):

(1) $$A(t)=A_{0}+\mu_{\max}F(t)-\ln\left(1+\left(\frac{\exp\left(\mu_{\max}F(t)\right)-1}{\exp\left(A_{m}-A_{0}\right)}\right)\right)$$

where A(t) is the concentration of cells (LogCFU·mL^−1^) or concentration of reducing sugars (%) at the time instant t; A_0_ is the minimum concentration of cells (LogCFU·mL^−1^) or minimum content of reducing sugars (%). A_m_ is the maximum concentration of cells (LogCFU·mL^−1^) or maximum content of reducing sugars (%); *μ*_max_ is the maximum rate of growth or substrate consumption (h^−1^). For the Baranyi model, the F(t) function is defined by Equation (2):

(2) $$F(t)=t+\frac{1}{v}\ln\left(\exp\left(-vt\right)+\exp\left(-h_{0}\right)-\exp(-vt-h_{0})\right)$$

where t is the time, $h_{0}$ = $\mu_{\max}\lambda$, and λ is the duration of the lag phase, v is the maximum rate of limiting substrate consumption, assumed to be equal to *μ*_max_ [21].

#### 2.2.2. Gompertz Model

The Gompertz model [22], with 3 parameters, is given according to Equation (3):

(3) $$A(t)=A_{m}\exp\left[-\exp\left(-\mu_{\max}\left(t-I\right)\right)\right]$$

where I is the time (h) at which the rate of growth or substrate consumption is maximum.

#### 2.2.3. Generalized Gompertz Model

The generalized Gompertz model [23], with 4 parameters, is given by Equation (4):

(4) $$A(t)=A_{0}+\left(A_{m}-A_{0}\right)\exp\left[-\exp\left(-\mu_{\max}\left(t-I\right)\right)\right]$$

#### 2.2.4. Logistic Model

The logistic model [24], with 3 parameters, is given according to Equation (5):

(5) $$A(t)=\frac{A_{m}}{1+\exp\left(-\mu_{\max}\left(t-I\right)\right)}$$

#### 2.2.5. Modified Logistic Model

The modified logistic model [25], with 4 parameters, is given by Equation (6):

(6) $$A(t)=A_{0}+\frac{A_{m}-A_{0}}{1+\exp\left(\frac{4\times \mu_{\max}\left(\lambda -t\right)}{A_{m}}+2\right)}$$

#### 2.2.6. Weibull Model

The Weibull model [26], with 4 parameters, is given by Equation (7):

(7) $$A(t)=A_{m}-\left(A_{m}-A_{0}\right)\exp\left[-{\left(k_{1}t\right)}^{\delta_{1}}\right]$$

where $k_{1}$, given in h^−1^, is the parameter that governs the rate at which the response variable approaches its maximum potential; and $\delta_{1}$ is an allometric constant. This constant is a parameter that controls the ordinate t (fermentation time) for the inflection point where the Weibull model is an exponential curve, if its value is equal to 1. However, for $\delta_{1}$ > 1, the Weibull model is a sigmoidal curve [23].

The parameters of the functions presented, as well as the statistical indicators, were determined by nonlinear regression using the least squares method, through the Levenberg–Marquardt algorithm.

### 2.3. Validation of Selected Models

The fitting of the selected primary models to the experimental data of the fermentation kinetics of *L. salivarius* was evaluated considering the coefficient of determination R^2^, chi-square $\chi^{2}$ and also the Student’s *t*-test for each parameter determined. In addition, the values predicted by the models were compared with the experimental values through the bias factor (BF) and accuracy factor (AF) tests [27,28], which are given according to Equations (8) and (9):

(8) $$\mathrm{BF}=10^{\frac{\sum_{i=1}^{n}\mathrm{Log}\left(\frac{\mathrm{Predicted}}{\mathrm{Experiment}}\right)}{n}}$$

(9) $$\mathrm{AF}=10^{\frac{\sum_{i=1}^{n}\left|\mathrm{Log}\left(\frac{\mathrm{Predicted}}{\mathrm{Experiment}}\right)\right|}{n}}$$

where n is the total number of experimental data.

The statistical indicator bias factor (BF) is a measure of the mean variation between the experimental value and the value predicted by the model. The accuracy factor (AF) measures the mean difference between the experimental values and the predicted values, disregarding whether the difference is positive or negative. A value close to 1 for BF and AF indicates that there is an agreement between the experimental and predicted values [14].

## 3. Results and Discussion

### 3.1. Optimization of Fermentation with L. salivarius

Table 2 summarizes the main results related to the optimization of fermentation with *L. salivarius*.

The growth of probiotic microorganisms such as *L. salivarius* in filmogenic solutions opens up possibilities for applications in a variety of food products. As examples, fresh fruits, minimally processed and/or dehydrated, as well as bakery products and fish can be mentioned. In these products, probiotic microorganisms add not only functional characteristics, but also extend their useful life. According to the results obtained, at the incubation temperature of 25 °C, *L. salivarius* showed a time interval of lag phase duration of approximately 2 h. This was the time without observation of significant increase in the number of cells, which corresponds to the period of adaptation of the microorganism in the culture medium. A period of approximately 2 h was also observed by Mis Solval et al. [29], when studying the growth kinetics of *Lactobacillus plantarum* NRRLB-4496, *Lactobacillus acidophilus* NRRLB-4495 and *Lactobacillus reuteri* B-1417 in a medium containing hydrolyzed egg white. Mustafa et al. [30] observed a short phase of adaptation of *Lactobacillus casei* in the fermentation of pomegranate (*Punica granatum*) juice. The growth curves of *L. salivarius* fermented at temperatures of 35 and 45 °C showed a short adaptation period (<2 h), which demonstrates a better adaptation of the microorganism to the filmogenic solutions under these fermentation conditions.

In the present study, the greatest growth of *L. salivarius* in gelatin- and inulin-based filmogenic solutions occurred when they were fermented at 45 °C, as there was an increase of approximately 2 LogCFU·mL^−1^ compared to the initial concentration of cells. At temperatures of 25 and 35 °C, there was an increase of approximately 1 LogCFU·mL^−1^ of the initial cell concentration. The cell production of *L. salivarius* in filmogenic solutions was inferior to the growth of other microorganisms of the same genus, *Lactobacillus*. As an example, Lin et al. [31] observed an increase of approximately 5 LogCFU·mL^−1^ of the initial cell concentration, when studying the growth of *Bifidobacterium* and *Lactobacillus* in soy milk and Man, Rogosa and Sharpe (MRS) medium. However, the development of probiotic microorganisms in different culture media is influenced by extrinsic and intrinsic factors of the system in which fermentation is carried out. Monteiro et al. [32], in a study on the growth of *L. reuteri* cells in passion fruit pulp, reported an increase in the initial number of cells of 1 LogCFU·mL^−1^, when the pulp was fermented at 35 °C. These researchers showed the importance of studying fermentation conditions and the limiting factor of the culture medium composition.

In view of the results obtained in the present study, it was estimated that the maximum concentration of cells was obtained after 4 h of fermentation; the higher concentrations were related to the experiments where fermentation was carried out at a temperature of 45 °C.

**Figure 1:**
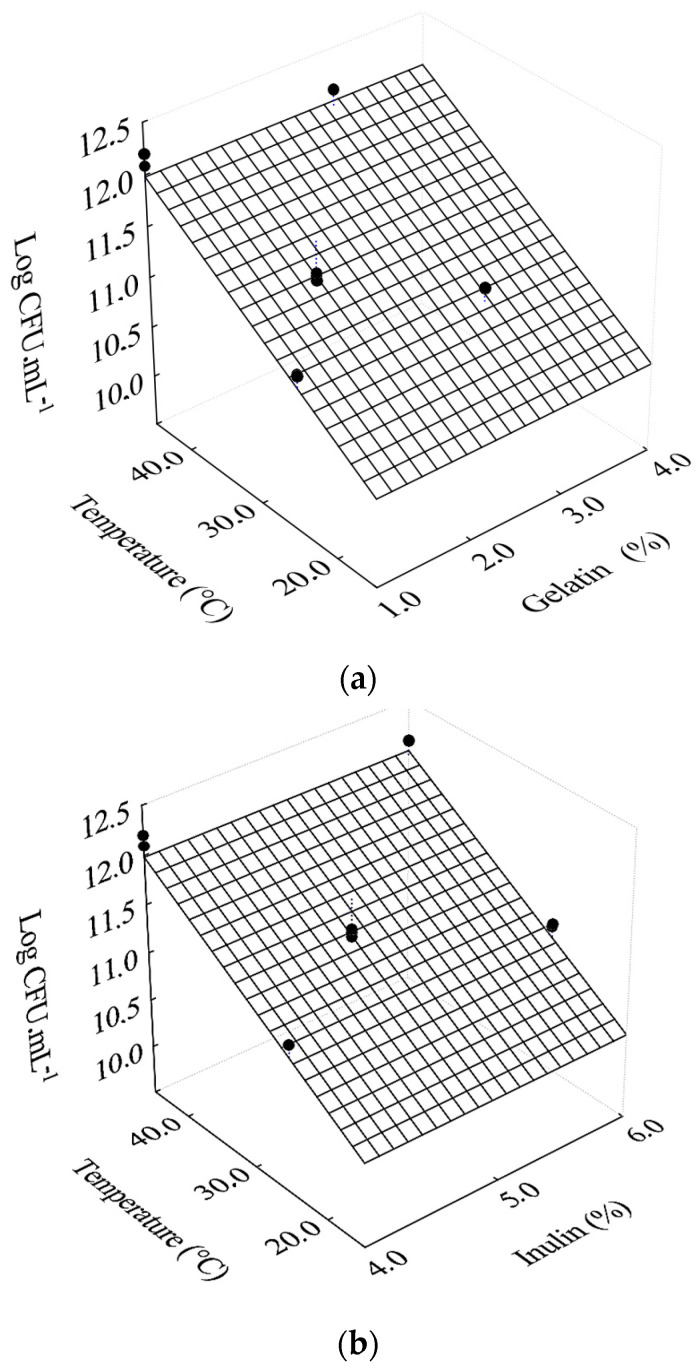
Response surfaces for the effects of temperature as a function of gelatin concentration (**a**) and inulin concentration (**b**) on cell concentration.

Table 2 summarizes the biological and physicochemical characteristics obtained from the experimental data after 4 h of fermentation of filmogenic solutions with *L. salivarius*. The effects of independent variables on fermentation with *L. salivarius* in filmogenic solutions showed that incubation temperature during fermentation influenced the population of cells in the filmogenic solutions, regardless of gelatin (Figure 1a) and inulin (Figure 1b) concentrations.

As suggested in Figure 1, temperature has a significant effect on the fermentation; probiotic cells reach a maximum number when the process occurs at 45 °C, which is the most indicated temperature for the growth of *L. salivarius*.

Equation (10) shows the statistically significant effect of temperature on the concentration of probiotic cells in the filmogenic solutions.

(10) $$\mathrm{Log}\mathrm{CFU}\cdot mL^{-1}=9.58+0.0532\times T$$

with R^2^ = 0.806, where LogCFU·mL^−1^ represents the maximum cell concentration, and T is the temperature (°C). Thus, by Equation (10), LogCFU·mL^−1^ varies between about 11 and 12, when the temperature ranges from 25 to 45 °C, respectively. Therefore, these results reasonably agree with the data in Table 2.

Equation (11) shows the model that describes the effect of temperature on the maximum growth rate of *L. salivarius* in the filmogenic solutions.

(11) $$\mu_{\max}=0.804-0.012\times T$$

with R^2^ = 0.716. Although the coefficient of determination of Equation (14) is low, this equation shows that the fermentation temperature significantly influences the maximum growth rate of *L. salivarius* in the filmogenic solutions. It is observed that, as the temperature increased from 25 to 45 °C, the maximum growth rate decreased.

**Figure 2:**
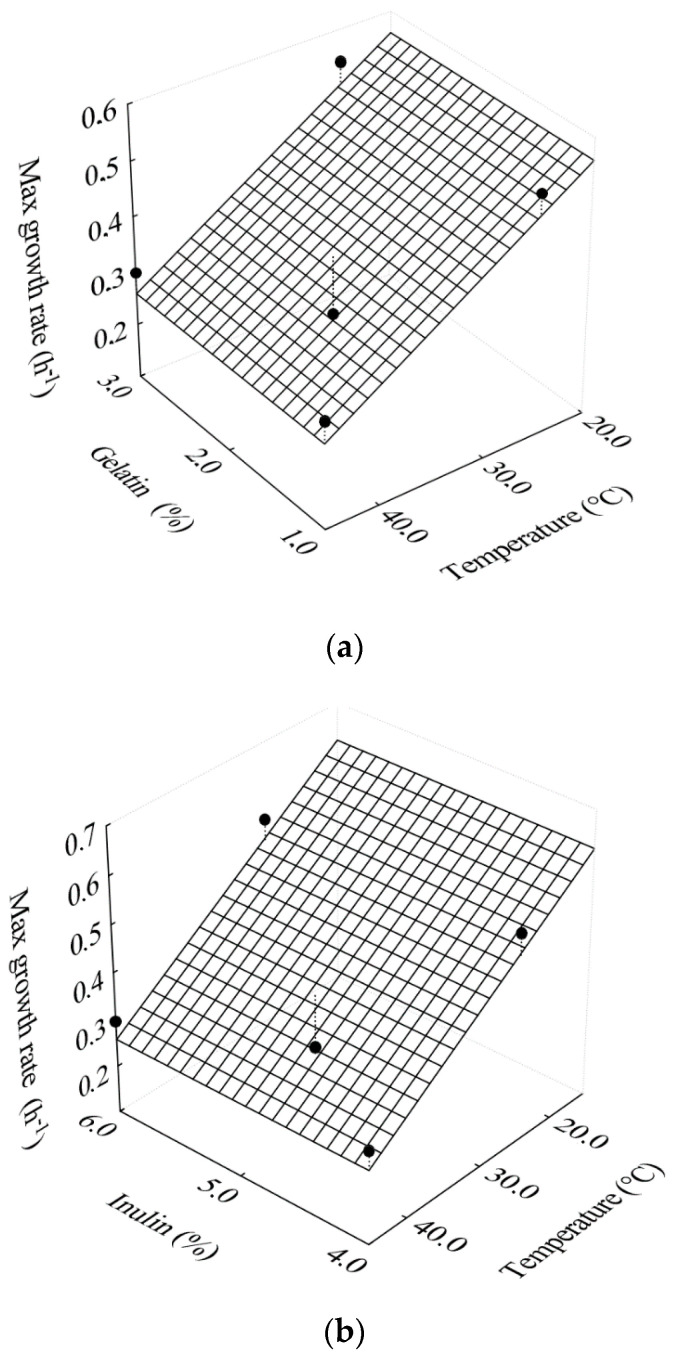
Response surfaces for the effects of temperature on the maximum cell growth rate as a function of (**a**) gelatin concentration; (**b**) inulin concentration.

Monteiro et al. [32], when studying the growth of *L. reuteri* in passion fruit pulp, observed that the maximum rate of cell growth ranged from 0.009 to 0.097 h^−1^, according to the influence of passion fruit pulp pH and fermentation temperature. Mestres et al. [33], studying the modeling of the mixed fermentation of gowé (Beninese fermented beverage made with malted and non-malted sorghum flour, which is produced by spontaneous fermentation involving mixed cultures of lactic acid bacteria and yeasts), using *L. plantarum* and *Pichia kluyveri* strain, observed a maximum growth rate of *Pichia kluyveri* of 0.73 h^−1^, using the logistic model to determine this rate. On the other hand, Mechmeche et al. [34], when analyzing kinetics and studying mathematical modeling to determine growth parameters of *Lactobacillus plantarum* in protein-rich isolates from tomato seed, observed a maximum growth rate of 0.169 h^−1^ (in the protein isolate of tomato seeds) and 0.363 h^−1^ (in the MRS broth). The literature cited shows the influence of several factors on the growth rate of different microorganisms. In the present study, Table 2 showed a growth rate variation of 0.27 to 0.54 h^−1^ in the experiments carried out at temperatures of 35 and 25 °C, respectively. This effect is observed in Figure 2a,b.

Under ideal conditions, bacteria are the microorganisms with the highest growth rate and may have a generation time (t_g_) of less than 1 h [32]. The generation time, which is the parameter that indicates the time required for the duplication of the cell population, ranged from 1.28 to 2.56 h in the present study. Generation time and pH were not statistically significant for the effects of independent variables on the fermentation process of filmogenic solutions.

**Figure 3:**
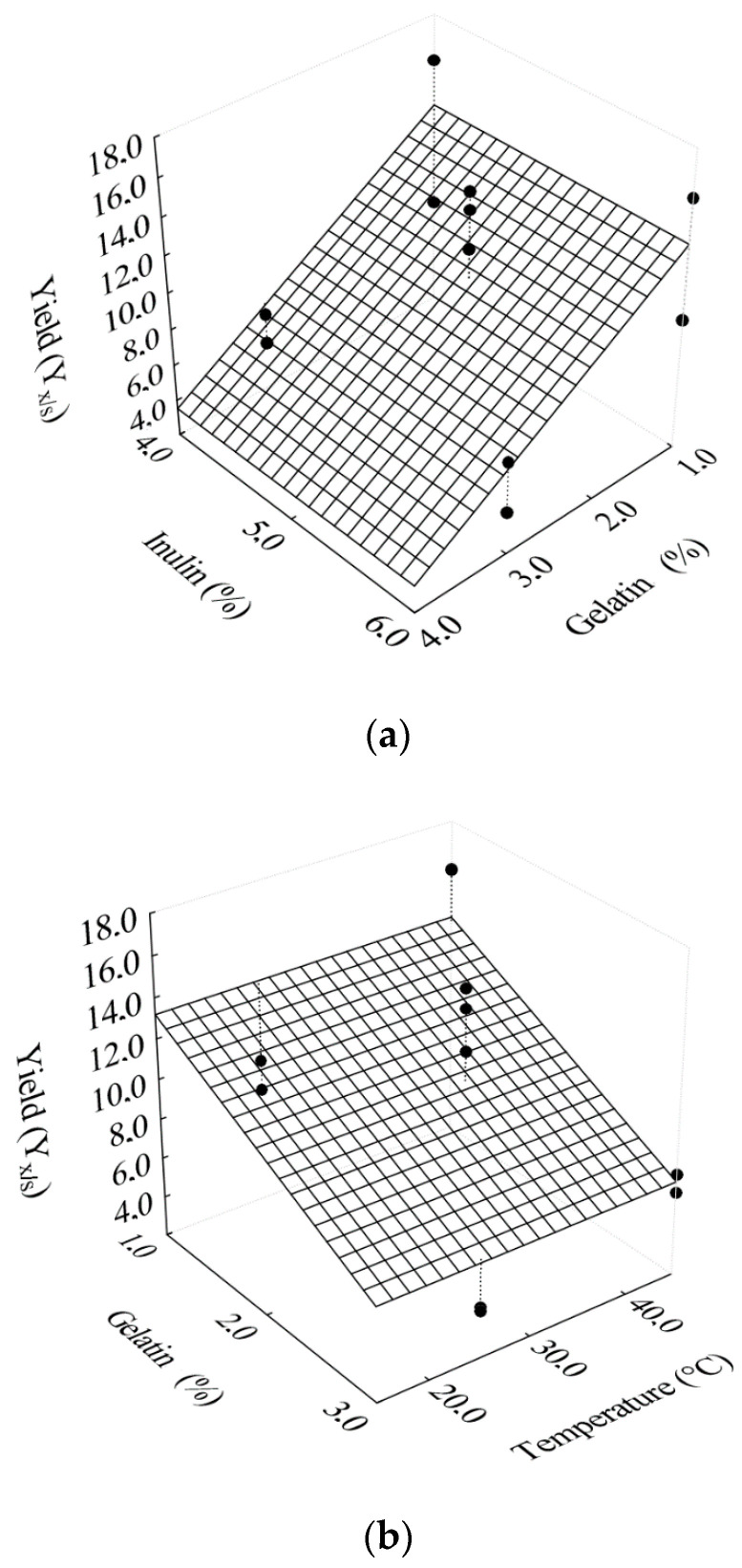
Response surfaces for the effects of gelatin concentration on cell yield as a function of (**a**) inulin; (**b**) temperature.

Cell yield $Y_{X/S}$, which represents the cell mass produced by the amount of substrate consumed, had the effect of gelatin concentration, since the increase in gelatin concentration in filmogenic solutions resulted in a reduction in cell yield (Figure 3a,b).

The solutions with 3% gelatin in the formulation resulted in more visually consistent filmogenic solutions, approaching the semi-solid state, which can become a barrier for the growth of the microorganism. The model with statistically significant regression coefficients is presented in Equation (12). The influence of gelatin on the model representing cell yield obtained a coefficient of determination of 0.852, but after parameterization, the coefficient of determination decreased to 0.653. Although parameterization has resulted in a reduction in the coefficient of determination, this resource was adopted in order to eliminate the effects of non-significant parameters for cell yield.

(12) $$Y_{X/S}=12.432-0.068\times G$$

with R^2^ = 0.653, where G represents the gelatin concentration in the filmogenic solutions (%). Although the function presented by Equation (12) has a very low coefficient of determination and is not useful for predicting cell yield, both this equation and Figure 3 show a tendency of the behavior of the cell yield as related to the gelatin concentration.

Physicochemical parameters of filmogenic solutions were determined in order to evaluate the potential of these solutions for the incorporation of probiotics into foods (Table 2). The pH is one of the main factors influencing the growth of microorganisms. Initially, the pH values of the solutions were between 5.6 and 5.9, and the pH variation may have been influenced by composition. After 4 h of fermentation, the filmogenic solutions had pH between 5.4 and 5.9 (Table 2). *L. salivarius* grows at optimum pH within the range from 5.5 to 6.5 [35]. It is worth pointing out that the filmogenic solutions based on gelatin and inulin have pH values within the range indicated as ideal, enabling the good development of *L. salivarius*.

According to Table 2, after 4 h of fermentation, soluble solids varied according to the concentration of gelatin and inulin. Soluble solids are composed largely of sugars and organic acids, as well as other constituents. Soluble solids are an important response of the fermentation process, and their quantity is influenced by the consumption of sugars by the microorganism and production of organic acids, such as lactic acid.

The concentration of reducing sugars was quantified in the filmogenic solutions, after 4 h of fermentation, and values between 1.06 and 1.71% were obtained, according to Table 2. Reducing sugars are simple sugars, and their presence in filmogenic solutions favors the development of lactic acid bacteria, which use simple sugars as the main source of energy. These values are different from the amount of sugars found in milk (4.49 g of lactose/100 g) [36] and in fruits such as passion fruit (4.86 g of glucose/100 g) [32], which have been studied for the production of probiotic foods. However, inulin is a widely studied prebiotic that acts as a substrate for the development of microorganisms, enabling a favorable environment for the growth of *L. salivarius* in filmogenic solutions.

**Figure 4:**
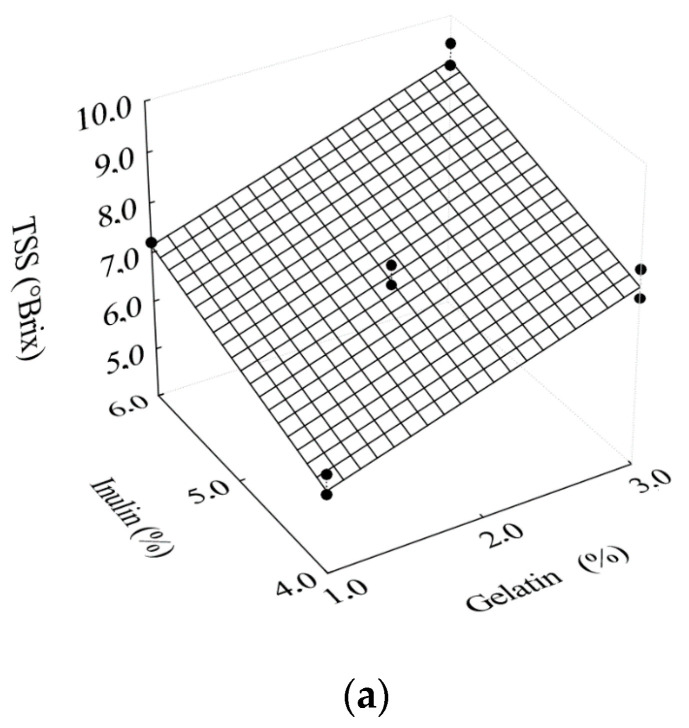

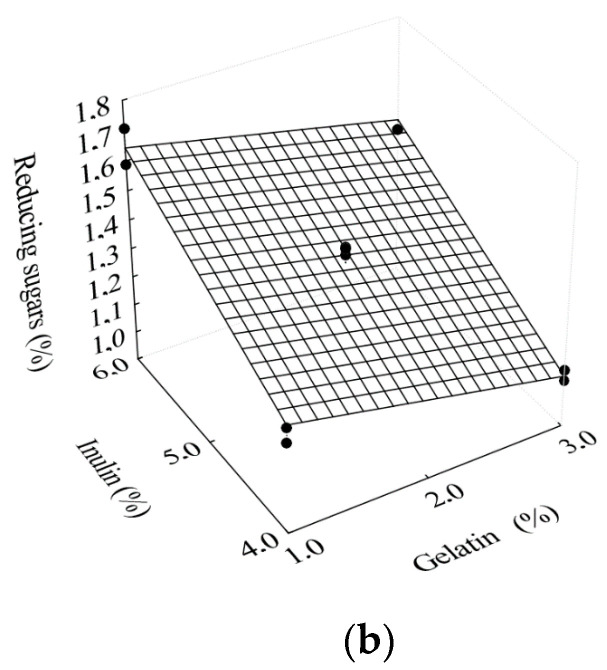
Response surfaces for the effects of gelatin and inulin concentrations as a function of (**a**) total soluble solids; (**b**) reducing sugars.

The concentrations of inulin and gelatin in the filmogenic solutions resulted in significant variations in total soluble solids and reducing sugars (Figure 4a,b).

As can be seen in Figure 4a, the increase in inulin and gelatin concentrations led to the increase in total soluble solids. On the other hand, it is interesting to note in Figure 4b that the highest content of reducing sugars was obtained for the highest inulin concentration combined with the lowest gelatin concentration. Equations (13) and (14) show polynomial models with only statistically significant regression coefficients for the effects of gelatin and inulin concentrations on total soluble solids and reducing sugars, respectively.

(13) TSS=6.4972+0.0145×G+0.0098×In

(14) RS=1.3373−0.0024×G+0.0031×In

with R^2^ = 0.763 and R^2^ = 0.889, respectively. In Equations (13) and (14), RS represents the content of reducing sugars (%), In is the concentration of inulin (%) and TSS represents total soluble solids (°Brix).

The study of gelatin and inulin concentrations, as well as the fermentation temperature of filmogenic solutions, through the response surface methodology, promoted the optimization of the fermentation process with *L. salivarius*. The results obtained showed that the most indicated conditions for the elaboration of probiotic filmogenic solutions were as follows: 1% gelatin, 4% inulin and fermentation at 45 °C (experiment 5). These conditions were defined according to the responses obtained for the physicochemical and biological characteristics of the probiotic filmogenic solutions that were statistically significant for, at least, one independent variable. The filmogenic solution obtained in experiment 5, initially presented a reducing sugar content of 1.35% and after 4 h of fermentation the reducing sugar content of the solution was 1.22% (Table 2), which means that in 4 h of fermentation approximately 9.6% of the reducing sugars present in the solution were consumed by *L. salivarius*. According to Ren et al. [37], the highest metabolic activity for the *L. salivarius* strain was observed when studying the use of maltose, raffinose, sucrose and glucose as preferred substrates. In this study, inulin-type fructan was used as a substrate. The temperature of 45 °C was selected based on the effect on cell concentration, and the highest growth was obtained in the experiments whose process occurred at this temperature. In addition, the concentrations of 1% gelatin and 4% inulin were the ones that most contributed to cell yield and correspond to experiment 5. Thus, the data of this experiment related to the kinetics of cell growth and substrate consumption were used in the following mathematical modeling.

### 3.2. Mathematical Modeling: Cell Growth and Substrate Consumption

Mathematical modeling in a fermentation process plays an important role, due to the guarantee of process control, economic production and increased product quality, since mathematical models are used to describe the production process under different fermentation conditions [15]. The mathematical models used in this study were selected based on the literature and are often used to describe both cell growth and substrate consumption. The Baranyi model, as well as the Gompertz, logistic and Weibull models, are some of the most used to predict experimental data and parameters of biological significance of fermentations.

#### 3.2.1. Cell Growth

The experimental data to be analyzed refer to the curve of cell growth in the filmogenic solution and were obtained under the following conditions: 1% gelatin, 4% inulin at 45 °C (experiment 5). The results of the fitting of the selected functions to the experimental data are presented in Table 3.

An inspection in Table 3 makes it possible to state that the Baranyi, Gompertz, generalized Gompertz, logistic, modified logistic and Weibull models had coefficients of determination greater than 0.920, low chi-squares, and bias factor and accuracy factor equal or very close to 1, indicating a good agreement between the experimental data and the corresponding values predicted by the analyzed models. However, some of the parameters determined for these models did not show statistical significance, according to the results obtained by the Student’s *t*-test. As it is known, Student’s *t*-test is used in curve fitting to calculate the probability of a parameter being zero, despite the value determined in the fit.

The results of Table 3 indicate that, for the Baranyi model, the parameters $\mu_{\max}$ and λ have a 100% probability of being zero, despite the values determined in the fitting. Thus, as these parameters have no statistical significance, the Baranyi model was rejected to represent the cell growth data under study. However, it is important to note that Costa et al. [38], Martins et al. [39] and Tarlak et al. [14] used in their works the Baranyi model to estimate parameters of biological significance relative to the (1) growth of *Lactobacillus* and its inhibitory capacity against *Listeria monocytogenes* in culture medium; (2) quantification of lactic acid bacteria in samples of cooked meat in vacuum packaging; (3) growth kinetics of *Pseudomonas* spp. on button mushrooms (*Agaricus bisporus*) under isothermal and non-isothermal conditions, respectively. By fitting the Baranyi model to experimental data, these authors successfully estimated the maximum cell growth rate, the duration of the lag phase and the maximum cell population.

Table 3 also indicated that the Gompertz model has two parameters ($\mu_{\max}$ and I) with probabilities of about 2% of being zero. For the generalized Gompertz model, the probabilities related to the parameters $\mu_{\max}$ and I are 100%, and therefore, these two models (Gompertz and generalized Gompertz) were also rejected in the prediction of growth data in the present article. On the other hand, the Gompertz and generalized Gompertz models were used by Germec et al. [15] to fit cell growth, product formation, and sugar consumption of batch lactic acid (LA) fermentation in stirred tank bioreactor with carob extract. According to the authors, these models showed a good fit to the biomass production data, with coefficients of determination close to 1.00 for both models.

By re-inspecting Table 3, it can be noted that the modified logistic and logistic models should be rejected because some of their parameters have no statistical significance. It should be pointed out, however, that Munanga et al. [40] studied the modeling of lactic fermentation of gowé and obtained a good fit of the logistic model to the experimental data of *Lactobacillus brevis* and *L. plantarum* growth during fermentation. The modified logistic model was also successfully used by Ilgin et al. [16] to describe fermentative processes in the production of inulinase from carob extract using *Aspergillus niger*. According to the authors, a good fit of this model to the experimental data was obtained, with a coefficient of determination of 0.988, and also with a bias factor and accuracy factor of 0.95 and 1.14, respectively.

In regard to the Weibull model, the information in Table 3 indicates that, although the parameter $k_{1}$ has a probability well below 1%, the parameter $\delta_{1}$ has a probability of about 71% of being zero, despite the value obtained in the fitting. Thus, the Weibull model was also rejected to represent the cell growth studied in this article.

Although the six models initially selected to describe cell growth kinetics have been successfully used in the literature, none of them could be recommended for this purpose in the present study. In this context, it is important to highlight the statement made by Silva et al. [41], citing van Boekel [42], that from a rigorous point of view, parameters obtained from experimental data without taking into account statistical indicators, in particular the Student’s *t*-test, can be considered uninterpretable.

Thus, the solution proposed in the present study was to use LAB Fit Curve Fitting Software (www.labfit.net) to discover another fitting function, taking advantage of a particular feature of this software program: LAB Fit has a library with more than 200 simple functions, with 1, 2, 3 and 4 parameters. More than that, LAB Fit has a feature called “Finder”, which makes it possible to fit all the functions of its library to a single set of experimental data, in a few seconds, and the best functions are chosen based on the lowest chi-squares obtained. Thus, by using the “Finder” and stipulating functions with up to three fitting parameters, the following result was obtained for the growth data: function numbers 81 (logistic, R^2^ = 0.9250), function number 80 (Gompertz, R^2^ = 0.9229) and function number 57, given by $Y=A+B\times \mathrm{EXP}\left(C\times X\right),R^{2}=0.9208$. As the first two of the three functions had been previously rejected, the third function was further investigated in an attempt to avoid non-significant parameters and to increase the coefficient of determination R^2^ by fitting this function to cell growth data.

Parameters A and B of function number 57 have physical interpretations related to cell concentrations, and parameter C is related to growth rate. Thus, these parameters were kept as they are, and only a possible change in exponent 1 was investigated, which can be explained in the expression $\mathrm{EXP}\left({\left(C\times X\right)}^{1}\right)$. This exponent 1, which defines an exponential function, could be, a priori, increased to 2 or 3, defining sigmoid functions. In a first investigation, the exponent was established with the value 2, instead of 1, and the coefficient of determination of the fit to the growth data increased from 0.9208 to 0.9710. In a second attempt, the value 3 was assigned to the exponent, and the fitting determination coefficient increased to 0.9813. However, in order to resist the temptation to further increase the exponent, it should be noted that $\mathrm{EXP}\left(-{\left(0.45\times 14\right)}^{2}\right)=5.78\times 10^{-18}$. Additionally, in terms of computational calculations, $\mathrm{EXP}\left(-{\left(0.45\times 14\right)}^{3}\right)=0.0$. It is worth pointing out that 0.45 h^−1^ and 14 h are typical values of growth rate and maximum experimental time in the present study. Thus, the exponent for the function proposed here was established with the value 3, and Statistica software was used again to fit function number 57 to the growth data, being rewritten as follows:

(15) $$A(t)=A_{m}-\left(A_{m}-A_{0}\right)\exp\left(-{\left(k_{1}t\right)}^{3}\right)$$

Table 4 shows the results regarding the fit of the proposed model, Equation (15), to the data of *L. salivarius* growth in the filmogenic solution.

The results obtained with the fit of the proposed model to the experimental data of cell growth showed that all three parameters have a 0% probability of being zero, and therefore, all parameters determined can be considered significant. Although simple, the results obtained showed that the proposed model was the most adequate to describe the growth of *L. salivarius* in the filmogenic solution during fermentation.

**Figure 5:**
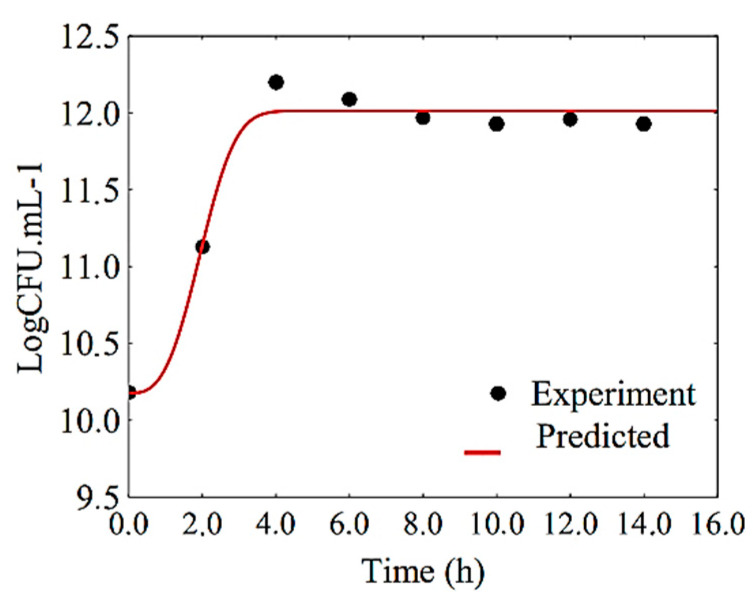
Curve of *L. salivarius* growth in the filmogenic solution represented by the proposed model.

Figure 5 shows the growth of *L. salivarius* in the filmogenic solution during fermentation. As can be observed, the parameters obtained resulted in a good fit of the proposed model to the experimental growth data. On the other hand, one should note that all phases of cell growth, except the decline phase, were satisfactorily simulated. The phase of decline or cell death began after 14 h of fermentation, and the data of this phase were not included in the mathematical modeling, since the present study did not aim to determine parameters that included this type of biological effect.

To complement the statistical information relative to the proposed model, the matrix of covariances, involving the parameters $a_{1}\equiv A_{m}$, $a_{2}\equiv A_{0}$ and $a_{3}\equiv k_{1}$ should also be informed:

(16) $$\mathrm{cov}=\left[\begin{array}{ccc} 2.06023\times 10^{-3} & 3.70901\times 10^{-5} & -2.75671\times 10^{-4}\\ 3.70901\times 10^{-5} & 1.22479\times 10^{-2} & -1.35868\times 10^{-3}\\ -2.75671\times 10^{-4} & -1.35868\times 10^{-3} & 8.47428\times 10^{-4} \end{array}\right]$$

Thus, the uncertainty of the value of function A(t), given by Equation (15), for any instant t, can be calculated by the general error propagation formula [43,44]:

(17) $$\sigma_{A(t)}=\sqrt{\sum_{j=1}^{3}\sum_{k=1}^{3}\frac{\partial A(t)}{\partial a_{j}}\frac{\partial A(t)}{\partial a_{k}}\mathrm{cov}(a_{j},a_{k})}$$

Thus, with the expression of A(t) given by Equation (15), in LogCFU·mL^−1^, and the uncertainty $\sigma_{A(t)}$ given by Equation (17), and also with the values obtained for the parameters given in Table 4, as well as the matrix of covariances given by Equation (16), the value of A(t) for a given instant t can be calculated for several moments.

#### 3.2.2. Substrate Consumption

The Baranyi, Gompertz and logistic models were tested to describe substrate consumption during fermentation but did not fit to the experimental data. The other models involved in this study were fitted to the experimental data of substrate consumption, and the results are summarized in Table 5.

The generalized Gompertz and modified logistic models had a coefficient of determination of 0.991, while the Weibull model showed a coefficient of determination of 0.995. However, the parameters A_m_, $\mu_{\max}$ and I of the generalized Gompertz model did not show statistical significance. Similarly, the parameters $\mu_{\max}$, A_m_ and λ of the modified logistic model have no statistical significance. Additionally, the parameters $k_{1}$ and $\delta_{1}$ of the Weibull model are also not significant. On the other hand, the model proposed in this article (which can ultimately be interpreted as a modification of the Weibull model in which $\delta_{1}$ was fixed with the value 3.0) had a coefficient of determination equal to 0.991, a chi-square of 0.000133, and bias factor and accuracy factor close to 1. For the proposed model, the probability of the parameter $k_{1}$ being zero, despite having the obtained value, is only 0.1%. Thus, of all the results obtained in Table 5, the model proposed in this article can be considered as the most acceptable to describe the substrate consumption in the filmogenic solution during fermentation with *L. salivarius*.

**Figure 6:**
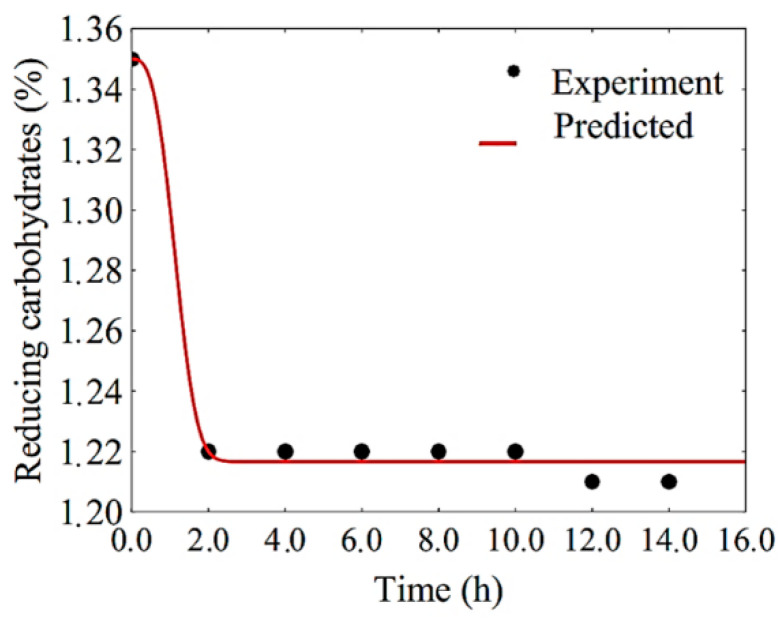
Substrate consumption curve during fermentation with *L. salivarius* in the filmogenic solution with fitting of the proposed model.

Reducing carbohydrates are consumed by *L. salivarius* during the fermentation process. In the first hours of fermentation, a sharp reduction was observed in this amount, as shown in Figure 6, which corresponds to the period of exponential growth of cell concentration in the filmogenic solution.

As can be seen in Figure 6, the concentration of reducing carbohydrates has its value stabilized between 2 and 4 h of the process. On the other hand, the decrease in the concentration of these carbohydrates occurs during the fermentation of the filmogenic solution, which is expected because the main metabolic process of lactic bacteria, such as *L. salivarius*, uses monosaccharides such as fructose, glucose and lactose for the production of energy and, therefore, lactic acid [32].

It is interesting to note that this behavior was well simulated by the proposed model. On the other hand, the matrix of covariances obtained by fitting this model to the experimental data of substrate consumption are given by:

(18) $$\mathrm{cov}=\left[\begin{array}{ccc} 4.44444\times 10^{-6} & 1.32349\times 10^{-23} & 1.05832\times 10^{-4}\\ 1.32349\times 10^{-23} & 2.66667\times 10^{-5} & 1.62818\times 10^{-5}\\ 1.05832\times 10^{-4} & 1.62818\times 10^{-5} & 1.84358\times 10^{-2} \end{array}\right]$$

Therefore, this is all the information needed for complete determination of the amount of substrate (reducing sugars, %) over time. Using Equations (17) and (18), it is possible to observe that, for t ≥ 3.0 h, this amount is given by RS = (1.217 ± 0.006) %, with a confidence level of 95.4%.

## 4. Conclusions

The growth of *L. salivarius* in gelatin- and inulin-based filmogenic solution has proved to be a viable alternative for incorporating probiotic bacteria into various food products. The use of probiotic filmogenic solution in food contributes to the development of innovative products with functional characteristics. The thin layer formed on the product’s surface forms a barrier that controls the contact of the food with the external environment, reducing the transfer of mass, which leads to an increase in its useful life, through the conservation of its sensory and nutritional characteristics. Evaluating the impacts of process conditions on cell development is fundamental and a key factor in making the process applicable in the industry. This study showed that the optimal conditions for the development of probiotic cells in the filmogenic solution were 1% gelatin combined with 4% inulin, fermented at 45 °C.

The analysis performed through the Student’s *t*-test of the parameters obtained by fitting the primary models selected in this work and widely used in predictive microbiology showed the inadequacy of such models to describe cell growth and substrate consumption during fermentation of filmogenic solution with *L. salivarius*. A simple model was then proposed in this article, with three fitting parameters, which described well the production of cells (R^2^ = 0.981, χ^2^ = 0.061, BF = 1.000 and AF = 1.001) and substrate consumption (R^2^ = 0.991, χ^2^ = 0.000133, BF = 1.000 and AF = 1.001). In this new model, the Student’s *t*-test revealed that all three parameters obtained by curve fitting can be considered statistically significant.

## Figures and Tables

**Table 1:** Matrix of the full 2^3^ experimental plan with three replicates at the central point with actual values of the independent variables.

Experiment	Independent Variables		
	Gelatin (%)	Inulin (%)	Temperature (°C)
1	1.0	4.0	25.0
2	3.0	4.0	25.0
3	1.0	6.0	25.0
4	3.0	6.0	25.0
5	1.0	4.0	45.0
6	3.0	4.0	45.0
7	1.0	6.0	45.0
8	3.0	6.0	45.0
9	2.0	5.0	35.0
10	2.0	5.0	35.0
11	2.0	5.0	35.0

**Table 2:** Biological and physicochemical characteristics of the filmogenic solutions fermented with *L. salivarius*, after 4 h of fermentation.

Parameters	Unit	Experiments										
		1	2	3	4	5	6	7	8	9	10	11
Maximum cell concentration	LogCFU·mL^−1^	11.05	11.06	11.03	11.06	12.20	12.09	12.08	12.09	11.09	11.05	11.13
Maximum growth rate $\left(\mu_{\max}\right)$	h^−1^	0.54	0.54	0.54	0.54	0.30	0.30	0.30	0.30	0.27	0.27	0.27
Generation time	h	1.28	1.28	1.28	1.28	2.31	2.34	2.34	2.34	2.54	2.56	2.54
Cell yield $\left(Y_{X/S}\right)$	LogCFU·mL^−1^ g^−1^	7.69	4.56	9.17	4.36	15.59	6.20	15.56	7.11	13.48	14.45	11.38
pH		5.63	5.47	5.53	5.47	5.40	5.53	5.43	5.43	5.77	5.90	5.80
Total soluble solids	°Brix	6.00	7.97	7.20	9.43	5.60	7.40	7.20	9.00	7.00	7.00	7.40
Reducing sugars	%	1.27	1.10	1.71	1.41	1.22	1.06	1.59	1.41	1.39	1.42	1.41

**Table 3:** Parameters of the models for the cell growth process in the filmogenic solution.

**Parameters**	**Baranyi Model**						
	**Estimated**	**t-Value**	***p*-Level**	**R^2^**	$\chi^{2}$	**BF**	**AF**
A_0_ (LogCFU·mL^−1^)	10.180 ± 0.100	83.585	0.000	0.982	0.059	1.000	1.001
A_m_ (LogCFU·mL^−1^)	12.013 ± 0.100	204.763	0.000				
$\mu_{\max}$ (h^−1^)	9.342 ± 883,919.2	0.000	1.000				
λ (h)	1.894 ± 10,199.1	0.000	1.000				
**Parameters**	**Gompertz Model**						
	**Estimated**	**t-Value**	***p*-Level**	**R^2^**	$\chi^{2}$	**BF**	**AF**
A_m_ (LogCFU·mL^−1^)	12.024 ± 0.107	112.864	0.000	0.923	0.253	1.000	1.003
$\mu_{\max}$ (h^−1^)	0.583 ± 0.171	3.416	0.019				
I (h)	−3.025 ± 0.936	−3.233	0.023				
**Parameters**	**Generalized Gompertz Model**						
	**Estimated**	**t-Value**	***p*-Level**	**R^2^**	$\chi^{2}$	**BF**	**AF**
A_0_ (LogCFU·mL^−1^)	10.180 ± 0.000	83.5850	0.000	0.982	0.059	1.000	1.001
A_m_ (LogCFU·mL^−1^)	12.013 ± 0.000	220.555	0.000				
$\mu_{\max}$ (h^−1^)	9.211± 5,247,461	0.000	1.000				
I (h)	1.954 ± 26,231	0.000	1.000				
**Parameters**	**Logistic Model**						
	**Estimated**	**t-Value**	***p*-Level**	**R^2^**	$\chi^{2}$	**BF**	**AF**
A_m_ (LogCFU·mL^−1^)	12.024 ± 0.104	116.106	0.000	0.925	0.246	1.000	1.003
$\mu_{\max}$ (h^−1^)	0.608 ± 0.167	3.633	0.015				
I (h)	−2.755 ± 0.822	−3.354	0.020				
**Parameters**	**Modified Logistic Model**						
	**Estimated**	**t-Value**	***p*-Level**	**R^2^**	$\chi^{2}$	**BF**	**AF**
A_0_ (LogCFU·mL^−1^)	10.180 ± 0.200	51.295	0.000	0.982	0.059	1.000	1.001
A_m_ (LogCFU·mL^−1^)	12.013 ± 0.100	220.562	0.000				
$\mu_{\max}$ (h^−1^)	23.910 ± 976,321	0.000	1.000				
λ (h)	1.740 ± 10,631.1	0.000	1.000				
**Parameters**	**Weibull Model**						
	**Estimated**	**t-Value**	***p*-Level**	**R^2^**	$\chi^{2}$	**BF**	**AF**
A_0_ (LogCFU·mL^−1^)	10.180 ± 0.122	83.733	0.000	0.982	0.059	1.000	1.001
A_m_ (LogCFU·mL^−1^)	12.013 ± 0.050	241.333	0.000				
$k_{1}$	0.470 ± 0.072	6.468	0.003				
$\delta_{1}$	5.018 ± 12.417	0.404	0.707				

A_0_ = minimum concentration of cells; A_m_ = maximum concentration of cells; AF = accuracy factor; BF = bias factor; I = time at which growth rate; *k*_1_ = rate at which the response variable approaches its maximum potential; R^2^ = coefficient of determination; *δ*_1_ = allometric constant; *λ* = duration of lag phase e *μ*_max_ = maximum growth rate; $\chi^{2}$ = chi-square.

**Table 4:** Parameters of the proposed model for the cell growth process in the filmogenic solution.

Parameters	Proposed Model						
	Estimated	t-Value	*p*-Level	R^2^	$\chi^{2}$	BF	AF
A_0_ (LogUFC.mL^−1^)	10.18 ± 0.11	264.668	0.000	0.981	0.061	1.000	1.001
A_m_ (LogUFC.mL^−1^)	12.013 ± 0.045	91.953	0.000				
$k_{1}$	0.453 ± 0.029	15.548	0.000				

A_0_ = minimum concentration of cells; A_m_ = maximum concentration of cells e *k*_1_ = rate at which the response variable approaches its maximum potential.

**Table 5:** Parameters of the models for the substrate consumption process in the filmogenic solution.

**Parameters**	**Generalized Gompertz Model**						
	**Estimated**	**t-Value**	***p*-Level**	**R^2^**	$\chi^{2}$	**BF**	**AF**
A_0_ (LogCFU·mL^−1^)	1.217 ± 0.003	467.281	0.000	0.991	0.000133	1.000	1.001
A_m_ (LogCFU·mL^−1^)	1.986 ± 565.278	0.004	0.997				
$\mu_{\max}$ (h^−1^)	1.860 ± 37.87	0.049	0.963				
I (h)	−0.89 ± 460.658	−0.002	0.999				
**Parameters**	**Modified Logistic Model**						
	**Estimated**	**t-Value**	***p*-Level**	**R^2^**	$\chi^{2}$	**BF**	**AF**
A_0_ (LogCFU·mL^−1^)	1.217 ± 0.003	466.927	0.000	0.991	0.000133	1.000	1.001
A_m_ (LogCFU·mL^−1^)	3.734 ± 3248.14	0.001	0.999				
$\mu_{\max}$ (h^−1^)	0.560 ± 11.056	0.051	0.962				
λ (h)	−2.655± 803.996	−0.003	0.998				
**Parameters**	**Weibull Model**						
	**Estimated**	**t-Value**	***p*-Level**	**R^2^**	$\chi^{2}$	**BF**	**AF**
A_0_ (LogCFU·mL^−1^)	1.110 ± 0.025	43.735	0.000	0.995	0.000082	1.000	1.001
A_m_ (LogCFU·mL^−1^)	1.350 ± 0.005	291.934	0.000				
$k_{1}$	0.003 ± 0.016	0.201	0.851				
$\delta_{1}$	0.054 ± 0.040	1.354	0.247				
**Parameters**	**Proposed Model**						
	**Estimated**	**t-Value**	***p*-Level**	**R^2^**	$\chi^{2}$	**BF**	**AF**
A_0_ (LogCFU·mL^−1^)	1.217 ± 0.002	577.116	0.000	0.991	0.000133	1.000	1.001
A_m_ (LogCFU·mL^−1^)	1.350 ± 0.005	261.426	0.000				
$k_{1}$	0.773 ± 0.116	6.635	0.001				

A_0_ = minimum concentration of substrate; A_m_ = maximum concentration of substrate; I = time at which rate substrate consumption is maximum; *k*_1_ = rate at which the response variable approaches its maximum potential; *δ*_1_ = allometric constant; *λ* = duration of lag phase e *μ*_max_ = maximum substrate consumption rate.
